# Somato-axodendritic release of oxytocin into the brain due to calcium amplification is essential for social memory

**DOI:** 10.1007/s12576-015-0425-0

**Published:** 2015-11-19

**Authors:** Haruhiro Higashida

**Affiliations:** Department of Basic Research on Social Recognition, Research Center for Child Mental Development, Kanazawa University, Kanazawa, 920-8640 Japan; Division of Socio-Cognitive-Neuroscience, Osaka University United Graduate School for Child, The Kanazawa Branchi, Takara-machi 13-1, Kanazawa, 920-8640 Japan

**Keywords:** Oxytocin, Hypothalamus, Social behavior, CD38, TRPM2

## Abstract

Oxytocin (OT) is released into the brain from the cell soma, axons, and dendrites of neurosecretory cells in the hypothalamus. Locally released OT can activate OT receptors, form inositol-1,4,5-trisphosphate and elevate intracellular free calcium (Ca^2+^) concentrations [(Ca^2+^)_*i*_] in self and neighboring neurons in the hypothalamus, resulting in further OT release: i.e., autocrine or paracrine systems of OT-induced OT release. CD38-dependent cyclic ADP-ribose (cADPR) is also involved in this autoregulation by elevating [Ca^2+^]_*i*_ via Ca^2+^ mobilization through ryanodine receptors on intracellular Ca^2+^ pools that are sensitive to both Ca^2+^ and cADPR. In addition, it has recently been reported that heat stimulation and hyperthermia enhance [Ca^2+^]_*i*_ increases by Ca^2+^ influx, probably through TRPM2 cation channels, suggesting that cADPR and TRPM2 molecules act as Ca^2+^ signal amplifiers. Thus, OT release is not simply due to depolarization–secretion coupling. Both of these molecules play critical roles not only during labor and milk ejection in reproductive females, but also during social behavior in daily life in both genders. This was clearly demonstrated in CD38 knockout mice in that social behavior was impaired by reduction of [Ca^2+^]_*i*_ elevation and subsequent OT secretion. Evidence for the associations of CD38 with social behavior and psychiatric disorder is discussed, especially in subjects with autism spectrum disorder.

## Introduction

Oxytocin (OT) and arginine vasopressin (AVP) are nonapeptides that differ in two amino acid residues [1]. OT and AVP are synthesized mostly in distinct neurons in the paraventricular nucleus (PVN) and supraoptic nucleus (SON) in the hypothalamus [2, 3]. OT and AVP are secreted into the blood circulation and have physiological roles in peripheral organs, such as the uterus, mammary gland, and kidney. They induce contraction of uterine and mammary duct smooth muscle or diuretic action in the kidney as hormones [4–6].

OT, AVP, and their receptors are present in the brain not only in females during specific reproductive periods but also in non-reproductive females and males [6]. Accumulating evidence has established that, in addition to classical hormonal functions, both peptides play critical roles in social recognition and social behavior in mammals, including humans [7–20]. This review focuses mainly on OT. The main point is not a general functional role of OT in a comprehensive review, but the molecular mechanisms of OT secretion into the brain that is critical in the neuronal function of OT in social recognition and behavior [4, 11, 13, 21].

Another reason to focus on the release is that the mechanism contains a very important aspect in terms of physiological science, in that the proposed idea challenges the principal rule in physiology of depolarization–secretion coupling [22–24]. Furthermore, this mechanism seems to have a potential relationship to autism spectrum disorder (ASD), a serious developmental disorder, which is a rapidly advancing field in neuroscience and psychiatry and is a serious disorder in our society [25–28]. There have been many reviews regarding the relationship between ASD and OT [29–35]. However, there have been few regarding the molecular mechanism of OT release into the brain [4], which is the critical step for social recognition and social behavior [26–28].

## Somato-axodendritic release of oxytocin

**Fig. 1:**
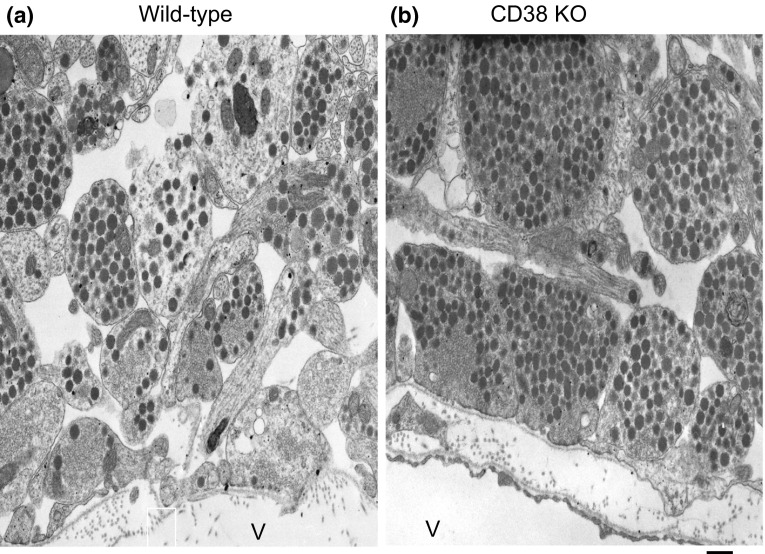
Electron micrographs of the posterior pituitary glands of wild-type (**a**) and CD38 knockout (**b**) mice. Vesicles are nerve endings close to the vascular space (V). Most of the dense core vesicles are oxytocinergic, as determined by immunoelectron microscopic examination. The nerve endings of CD38 knockout mice contain more vesicles than those of wild-type mice, indicating that vesicles are released in the wild-type mice and not secreted in CD38 knockout mice. *Bar* 500 nm (modified from Fig. 3 of Ref. [21])

OT is secreted from the nerve terminals of axons of oxytocinergic neurons at the perivascular site in the posterior lobe of the pituitary into the circulation [4] (Fig. 1). Oxytocinergic neurons send their axons to the amygdala and some other limited brain regions and secrete OT from the nerve terminals [4, 12, 15]. It is known that adrenaline stimulates oxytocinergic neurons in the SON, which results in local release of OT in the brain [5, 36]. This release occurs from the cell soma, axons, and dendrites, i.e., somato-axodendritic release [37–39].

**Fig. 2:**
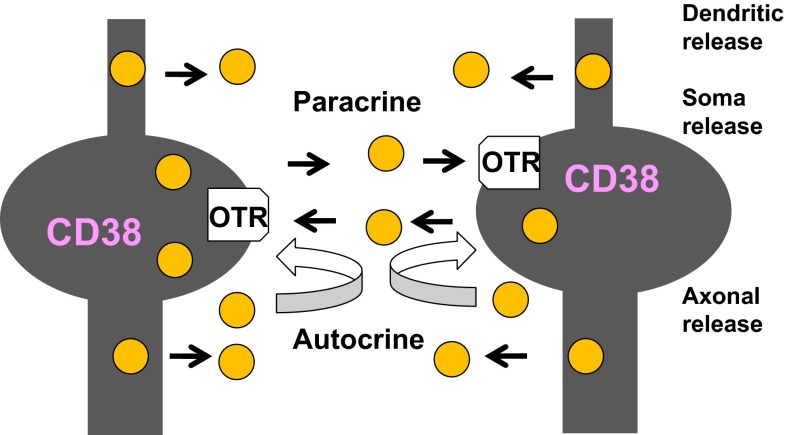
Scheme showing autocrine and paracrine release of oxytocin. OT is released from dendrites (dendritic release), from the cell soma (soma release), and from axons (axonal release) in the hypothalamus. Hypothalamic oxytocinergic neurons express OT receptors (OTR). Released OT binds to OTR. More OT (*yellow circle*) is released by CD38-mediated intracellular calcium amplification (not shown). The positive feedback of OT release occurs by OT released from self or nearby cells via autocrine and paracrine mechanisms, respectively

Locally released OT causes excitation of OT neurons by activating OT receptors expressed in neurons of both the PVN and SON [40–43]. OT stimulates OT receptors and facilitates OT release from the stimulated neurons. Released OT can stimulate OT receptors and elicits release from the same neurons (autocrine) or nearby neurons (paracrine) [44] (Fig. 2). This OT-induced OT release determines the basal brain concentrations and elevated concentrations of OT. The concept of autoregulation, OT-induced OT release, can be an extremely efficient way to achieve massive OT recruitment during uterine contraction in labor and milk ejection in lactation [5, 6, 45–47]. Autoregulation, however, is also an essential brain mechanism for social recognition in daily life in both genders, as proposed previously [25, 27, 28].

## Oxytocin receptors and cellular signaling

OT receptors are seven-transmembrane proteins that couple with the G_q/11_-type GTP-binding protein [48]. Stimulation of OT receptors leads to the production of inositol-1,4,5-trisphosphate (IP_3_) and diacylglycerol (DAG) through the activation of phospholipase C (PLC) [48]. This results in activation of Ca^2+^ mobilization from IP_3_-sensitive Ca^2+^ pools [49].

**Fig. 3:**
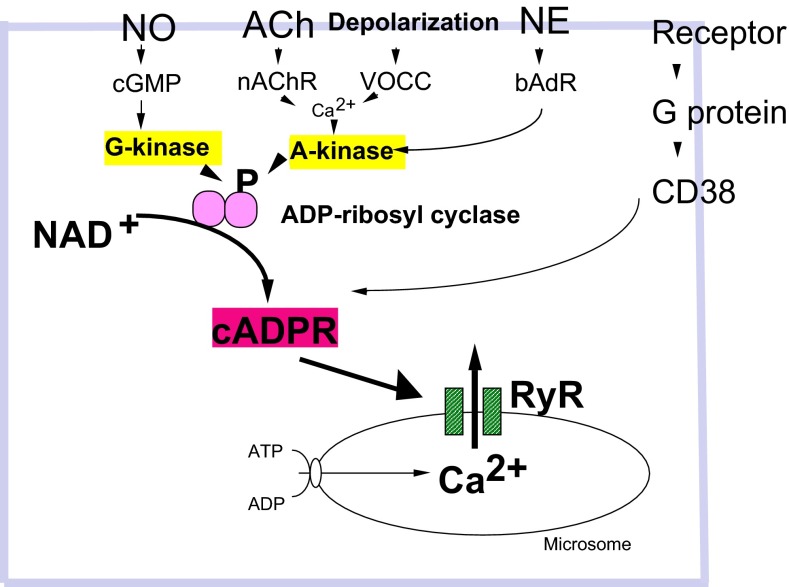
Intracellular signaling pathways leading to increased cyclic ADP-ribose formation. Phosphorylation (P) of ADP-ribosyl cyclase (*pink*) is mediated by several pathways. Nitric oxide (NO), cyclic GMP (cGMP), and protein kinase G (G-kinase); acetylcholine (ACh), nicotinic ACh receptors (nAChR); voltage-operated Ca^2+^ channels (VOCC), Ca^2+^ and protein kinase A (A-kinase); norepinephrine (NE), β adrenaline receptors (bAdR). Activation of CD38 by GTP-binding protein (G protein) and various types of receptors triggers formation of cADPR. cADPR opens Ca^2+^ release channels of ryanodine receptor type II or III (RyR) with another cofactor, Ca^2+^ (not shown). Mobilization of Ca^2+^ from microsomes of Ca^2+^ pools increases [Ca^2+^]_*i*_, resulting in OT release (not shown)

On the other hand, another Ca^2+^ signal pathway of cyclic ADP-ribose (cADPR) [50, 51] was identified downstream of OT receptors [11]. cADPR mobilizes Ca^2+^ through cADPR-sensitive Ca^2+^ pools, in a mechanism referred to as Ca^2+^-induced Ca^2+^ release. In this process, cADPR plays an essential role in mobilizing Ca^2+^ through Ca^2+^ channels of ryanodine receptors [52–56] (Fig. 3). The recent review by Leng et al. did not mention this cADPR/CD38 hypothesis [4], probably because they described by their data based on their finding with thapsigargin [36].

It is known that intracellular cADPR concentrations are regulated in many different ways, including activation of ADP-ribosyl cyclase or CD38, via heterotrimeric GTP-binding proteins, or phosphorylation downstream of the G protein-coupled receptor signaling pathways [57–59]. Specifically, the activation of ADP-ribosyl cyclase or CD38 by cyclic GMP- or cyclic AMP-dependent protein kinases has been reported in *Aplysia californica,* liver cells [60, 61], LAK cells [62, 63], and artery smooth muscle cells [57] (Fig. 3).

**Fig. 4:**
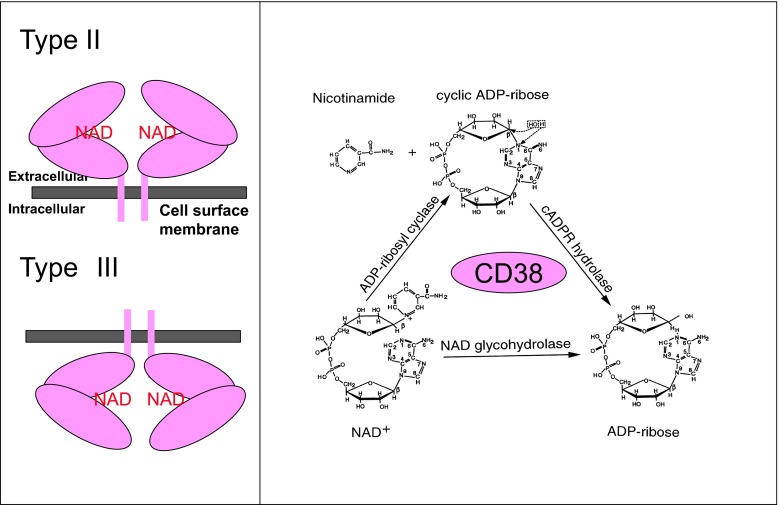
Membrane topology and enzyme reaction of CD38. CD38 (*pink oval*) usually forms a dimer. β-NAD^+^ binds to the central catalytic site of CD38. The large C-terminal part is located in the extracellular space, as the type II transmembrane protein, or intracellular space as the type III transmembrane protein, according to Lee and colleagues [66, 67]. CD38 has three enzymic activities. CD38 catalyzes formation of cyclic ADP-ribose from β-NAD^+^ by cleaving nicotinamide. cADPR is hydrolyzed to form ADP-ribose. β-NAD^+^ also has NAD^+^ glycohydrolase activity to form ADP-ribose from β-NAD^+^ in one step. The scheme of enzyme activity is modified from Lee [50]

cADPR is a catalytic product of ADP-ribosyl cyclase or ectopic CD38 [50, 51, 63] (Fig. 4). cADPR is produced in the extracellular space by the large C-terminal portion of CD38 with catalytic activity that may be present in the extracellular space. Therefore, it is unclear how extracellular cADPR produced by CD38 acts as an intracellular second messenger. It has been reported that cADPR applied extracellularly stimulates intracellular ryanodine receptors after internalization by the nucleotide-transporting capacity of CD38 in fibroblasts and astrocytes (the nucleotide carrier hypothesis of De Flora) [64, 65]. Recently, it was reported that the type II transmembrane glycoprotein, CD38, may exist in two forms with regard to membrane topology [66, 67]; the large C-terminal portion with catalytic activity may exist in the extracellular space as the type II protein, and this catalytic site may also exist inside the cell as the type III form (Fig. 4a). In the latter case, the product of CD38, cADPR, is produced intracellularly, and acts directly as a second messenger (two topology hypothesis of Lee).

## Effects of oxytocin on ADP-ribosyl cyclase and intracellular Ca^2+^ concentrations

Application of OT stimulates ADP-ribosyl cyclase activity or CD38 in crude membrane fractions, when measured by cADPR formation from β-NAD^+^ or by cyclic GDP-ribose (cGDPR) production from NGD^+^ [50, 68]. cADPR or cGDPR production increases in a concentration-dependent manner upon exposure to sub-nanomolar concentrations of OT [49].

Subsequently, in isolated hypothalamic neurons, application of 100 pM OT results in [Ca^2+^]_*i*_ increases: a rapid initial increase and a sustained elevation lasting for 5 min [69]. OT elicits an initial elevation of the maximum [Ca^2+^]_*i*_, and this phase is IP_3_-dependent. Pretreatment with 8-bromo-cADPR, an antagonist of the cADPR-binding site of Ca^2+^ release channels of ryanodine, inhibits OT-mediated sustained [Ca^2+^]_*i*_ increases. ADPR and β-NAD^+^ also induce elevation of [Ca^2+^]_*i*_ and replicate the second phase of sustained [Ca^2+^]_*i*_ increases [49, 69]. Under Ca^2+^-free conditions, the OT-mediated increase of [Ca^2+^]_*i*_ shows little change in either phase, suggesting that the two phases of [Ca^2+^]_*i*_ elevation in hypothalamic neurons are due to Ca^2+^ mobilization from the intracellular Ca^2+^ pools [49].

## Oxytocin release by extracellular application of cyclic ADP-ribose

**Fig. 5:**
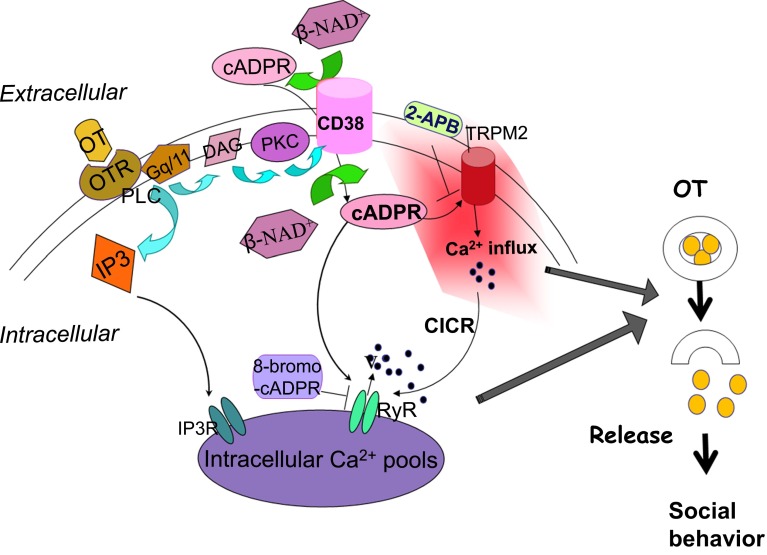
Oxytocin induced oxytocin release. Oxytocin (OT; *yellow circles*) stimulates oxytocin receptors (OTR). Subsequently, the G_q/11_ type G protein and phospholipase C (PLC) are activated, resulting in formation of inositol-1,4,5-trisphosphate (IP3) and diacylglycerol (DAG). Stimulated protein kinase C (PKC) activates CD38 and increases formation of cADPR from β-NAD^+^ inside or outside cells. cADPR activates Ca^2+^ influx TRPM2 cation channels. 2-Aminoethoxydiphenyl borate (2-APB) inhibits TRPM2 channels. IP3 induces mobilization of Ca^2+^. TRPM2 mediates Ca^2+^ influx, which also stimulates Ca^2+^ mobilization through ryanodine receptor Ca^2+^ release channels as a cofactor together with cADPR. These Ca^2+^ ions (*filled circles*) increased by Ca^2+^ amplification mechanisms stimulate OT (*yellow*) release into the brain, which is an essential step for social memory and social behavior. Modified from [11, 27, 52, 73]

High potassium-induced depolarization produces an increase of up to eightfold in OT secretion from isolated mouse hypothalamic neurons or their axon terminals in the posterior pituitary gland, respectively [21]. OT release is enhanced by about fourfold by application of extracellular β-NAD^+^, a precursor of cADPR (refer to Fig. 4 in [21]). The increase is blocked completely by 8-bromo-cADPR. To further confirm the involvement of cADPR, we examined the effects of extracellular application of several β-NAD^+^ metabolites [49, 69]. Only cADPR showed a potentiation effect, indicating that OT release utilizes the cADPR/ryanodine calcium amplification system (Fig. 5).

### Involvement of TRPM2 channels

Melastatin-related transient receptor potential channel 2 (TRPM2, previously named TRPC7 or LTRPC2) possesses ADPR hydrolase activity and is a Ca^2+^-permeable cation channel. β-NAD^+^, ADPR, and cADPR can activate TRPM2 channels [70]. TRPM2 activation by cADPR is promoted at body temperature (>35 °C) and is involved in insulin secretion in pancreatic β cells [71]. In addition, TRPM2 channels are related to receptor functions through cADPR formation [72].

**Fig. 6:**
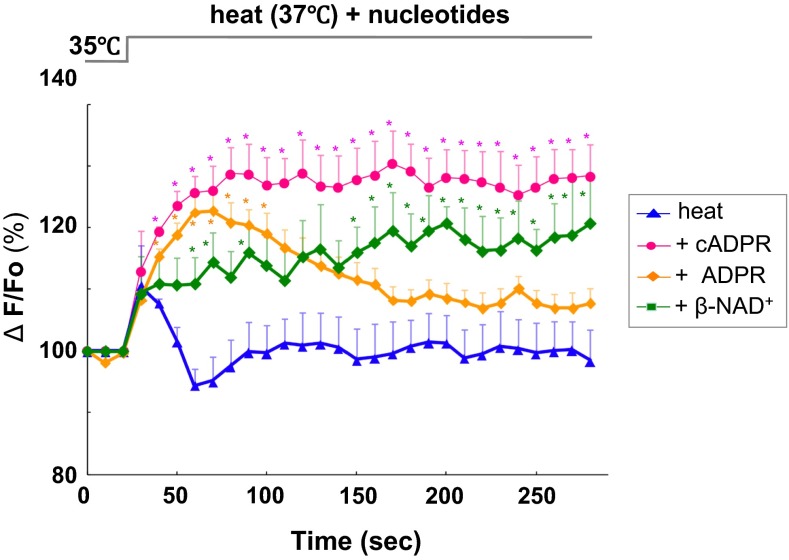
Effects of cyclic ADP-ribose, ADP-ribose, and β-NAD^+^ on heat-induced calcium concentration rise. Time course of [Ca^2+^]_*i*_ changes in Oregon Green-induced anterior hypothalamic neurons. At about 25 s after the beginning of each trace, cells were heated from 35 to 37 °C together with 100 μM cADPR, ADPR, β-NAD^+^ or without nucleotides (heat alone). Symbols indicate changes in [Ca^2+^]_*i*_ levels, represented by the fluorescence intensity at each time point relative to resting intensity at time zero. *N* = 3−5 experiments. Mean ± SEM. Modified from [69]

Extracellularly applied cADPR can activate [Ca^2+^]_*i*_ signaling via CD38 or TRPM2 channels downstream of OT receptors. [Ca^2+^]_*i*_ increases in the model neuron, NG108-15 mouse neuroblastoma × rat glioma hybrid cells that possess CD38 [58, 73] but not OT receptors [74], as in the isolated whole hypothalamus after stimulation with extracellularly applied cADPR [69, 75]. Interestingly, the same tissues show significantly greater increases upon extracellular challenge with cADPR together by heating to 40 °C from 35 °C in the incubation medium (Fig. 6). Little or no cADPR-mediated [Ca^2+^]_*i*_ elevation was observed at 40 °C in the absence of extracellular Ca^2+^. Ca^2+^ influx is expected, probably through non-selective cation TRPM2 channels, because elevation of [Ca^2+^]_*i*_ is inhibited by the TRPM2 channel inhibitor, 2-aminoethoxydiphenyl borate (2-APB). Similarly, 8-bromo-cADPR inhibits responses to β-NAD^+^ and heat. These results suggest that cADPR contributes to both Ca^2+^ mobilization from internal Ca^2+^ pools and Ca^2+^ influx through TRPM2 Ca^2+^-permeable channels from the extracellular space. Such [Ca^2+^]_*i*_ increases may result in OT release. However, there have been no previous reports regarding heat-induced OT release in the hypothalamus.

## Contribution of CD38

In the central nervous system, ADP-ribosyl cyclase activity corresponding to CD38 is detected as early as embryonic day 15 in mouse development [76]. In the brain, expression levels of CD38 and ADP-ribosyl cyclase activity increase with further development [77]. The role of CD38 in regulation of OT secretion through cADPR-mediated intracellular calcium signaling has been clearly demonstrated using CD38 knockout mice [11, 21, 78, 79]. The plasma and cerebrospinal fluid OT levels are reduced in CD38 knockout mice. Electron microscopic examination exhibited little to no release from the nerve endings of oxytocinergic neurons in the pituitary of CD38 knockout mice (Fig. 1). These phenotypes were rescued by simple subcutaneous injection of OT as well as brain local re-expression of human CD38, but not mutant CD38, by the lentivirus infection method in CD38 knockout mice [21].

## Human social behavior and psychiatric disorders

As CD38 is recognized as being closely related to OT release and social memory in mice, we examined the association of single nucleotide polymorphisms (SNPs) in the human CD38 gene on ASD [80]. In a series of elegant studies in 323 mothers, fathers, and non-parents, Epstein and colleagues reported that risk alleles on *CD38* (including rs3796863) genes are associated with less parental touch. In contrast, relatively high plasma OT levels in subjects with low-risk *CD38* alleles predict longer durations of parent–infant gaze synchrony. Furthermore, parents that display more touch toward their infants were reported to have been well cared for in childhood, to exhibit higher plasma to levels, and to have low-risk *CD38* alleles [29, 30, 81]. The mother’s *CD38* allele predicts parental behavioral synchrony at 1 and 6 months of their first-born infants and children’s social reciprocity during interactions with their best friend at 3 years. CD38 in the OT pathway was shown to be critical for parent–infant attachment and attention [82]. A SNP on the *CD38* gene is also associated with social integration and social connectedness [83].

Several studies indicated the association of *CD38* with ASD [84–87]. Ten SNPs and mutations of *CD38* were examined, and the *CD38* SNPs, rs6449197 and rs3796863, were shown to be linked with high-functioning ASD in participants in the USA but not in Japan. These findings were partially replicated among Israeli subjects [29, 31, 32, 87].

## Conclusion

**Fig. 7:**
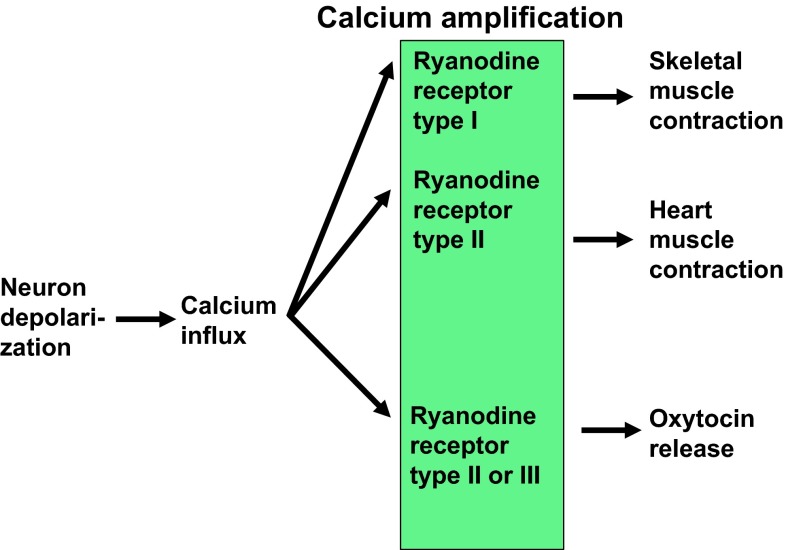
Scheme indicating Ca^2+^ amplification with different ryanodine receptor subtypes. Skeletal muscle contraction and heart muscle contraction utilize type I and II ryanodine receptors, respectively. Oxytocin release uses type II or III ryanodine receptors

This review discussed how OT is released into the brain. Ca^2+^ influx through Ca^2+^ channels is not sufficient to trigger OT release. The Ca^2+^ signal must be amplified by Ca^2+^-induced Ca^2+^ release through Ca^2+^ channels of ryanodine receptors type II or III by cADPR and some NAD metabolites in the hypothalamus (Fig. 7). In addition, Ca^2+^ influx through TRPM2 channels contribute more to increases in [Ca^2+^]_*i*_. This hypothesis of depolarization-independent but heat-sensitive Ca^2+^ signaling for OT release is consistent with the previous suggestion of dendritic release of OT without depolarization [4, 21, 39].

OT exerts an anxiolytic effect during stress, and stress sometimes induces hyperthermia. It is therefore interesting to examine how stress induces hyperthermia, which results in subsequent OT release. OT release seems to be important in damping the stress-induced disadvantage.

OT is an essential molecule for social memory and social behavior [21, 29]. Deficiency in social behavior is the core symptom of ASD. Recently, Yamasue and his group reported that repetitive intranasal OT administration for 6 weeks improved symptoms of the social behavior domain [88]. This result could be due to the delivery of OT to the brain by intranasal administration, but there is still little direct evidence regarding whether OT is recruited into the brain from the peripheral tissues or organs crossing the blood–brain barrier from the blood circulation. Several important questions regarding OT secretion into the brain and OT-induced Ca^2+^ signaling and OT transport from the blood to the brain remain to be resolved.
